# Multi-Stakeholder Taskforces in Bangladesh — A Distinctive Approach to Build Sustainable Tobacco Control Implementation

**DOI:** 10.3390/ijerph120100474

**Published:** 2015-01-07

**Authors:** Angela M. Jackson-Morris, Ishrat Chowdhury, Valerie Warner, Kayleigh Bleymann

**Affiliations:** Department of Tobacco Control, The International Union against Tuberculosis and Lung Disease, 8 Randolph Crescent, Edinburgh EH3 7TH, UK; E-Mails: ichowdhury@theunion.org (I.C.); vwarner@theunion.org (V.W.); kbleymann@theunion.org (K.B.)

**Keywords:** Bangladesh, tobacco control, sustainability, LMIC

## Abstract

The MPOWER policy package enables countries to implement effective, evidence-based strategies to address the threat posed to their population by tobacco. All countries have challenges to overcome when implementing tobacco control policy. Some are generic such as tobacco industry efforts to undermine and circumvent legislation; others are specific to national or local context. Various factors influence how successfully challenges are addressed, including the legal-political framework for enforcement, public and administrative attitudes towards the law, and whether policy implementation measures are undertaken. This paper examines District Tobacco Control Taskforces, a flexible policy mechanism developed in Bangladesh to support the implementation of the Smoking and Tobacco Products Usage (Control) Act 2005 and its 2013 Amendment. At the time of this study published research and/or data was not available and understanding about these structures, their role, contribution, limitations and potential, was limited. We consider Taskforce characteristics and suggest that the “package” comprises a distinctive tobacco control implementation model. Qualitative data is presented from interviews with key informants in ten districts with activated taskforces (n = 70) to provide insight from the perspectives of taskforce members and non-members. In all ten districts taskforces were seen as a crucial tool for tobacco control implementation. Where taskforces were perceived to be functioning well, current positive impacts were perceived, including reduced smoking in public places and tobacco advertising, and increased public awareness and political profile. In districts with less well established taskforces, interviewees believed in their taskforce’s ‘potential’ to deliver similar benefits once their functioning was improved. Recommendations to improve functioning and enhance impact were made. The distinctive taskforce concept and lessons from their development may provide other countries with a flexible local implementation model for tobacco control.

## 1. Introduction

The tobacco epidemic is having devastating impacts across the globe, with low- and middle-income countries (LMICs) facing a disproportionate burden. Currently 75% of tobacco users live in [1] and 80% of all tobacco-attributed deaths occur in LMICs [2]. Many of these countries are faced with rising affordability of tobacco products [3] and an aggressive tobacco industry that targets consumers and governments [4]. To combat the tobacco epidemic and its health and economic burdens, the World Health Organization (WHO) in 2008 introduced a package of six evidence-based measures, collectively known as “MPOWER”, to assist all countries to implement effective tobacco control. The components of MPOWER include: Monitor tobacco use and prevention policies; Protect people from tobacco smoke; Offer to help quit tobacco use; Warn about the dangers of tobacco; Enforce bans on tobacco advertising, promotion and sponsorship, and; Raise taxes on tobacco [2]. Implementation of the highest-level ‘MPOWER’ policies adopted between 2007 to 2010 is predicted to avert nearly 7.5 million smoking attributed deaths by the year 2050 [5]. Countries require robust legislation encompassing these measures and strong structures to enable authorities and communities to enforce these laws.

Inevitably, various obstacles and issues exist that need to be addressed to successfully implement tobacco control laws. Some obstacles to implementation—such as tobacco industry interference—are found in all countries [6] while there may be additional challenges that relate to each country’s particular legal, political, economic, and cultural context. For example, in China the government owns the China National Tobacco Corporation, a monopoly whose interests directly conflict with that of tobacco control and which adds legal, political and economic complexity to tobacco control implementation [7]. Whereas, a cultural issue requiring attention particularly in rural regions of India is the persisting popular belief that tobacco has medicinal value [8].

A number of different factors may then influence the extent to which these issues are addressed. The legal-political framework in which law enforcement takes place may allow for specific policy measures to be undertaken to assist implementation; for example, in Vietnam the national government issued three official Decrees that address National Tobacco Control Policy violations and individual ministries, including Health and Transport, have issued Directives outlining implementation measures in their respective sectors [9,10]. Public and administrative attitudes toward the law are also important. Anecdotal evidence suggests that in some countries there may be a tendency for public disregard towards laws on various issues and this may provide an additional challenge for tobacco control law implementation. In this paper, we focus on local-level tobacco control taskforces, a particular approach to assist tobacco control implementation in Bangladesh. We examine their role, functioning, strengths, weaknesses and potential contribution from the perspectives of a range of local stakeholders.

### Status and Characteristics of District Tobacco Control Taskforces in Bangladesh

Bangladesh is a low-income country with one of the world's highest rates of tobacco use; 43% of adults—over 41.3 million adult people—use tobacco [11]. Each year in Bangladesh, tobacco kills 57,000 people [12] and 1.2 million cases of illnesses are attributed to its use [13]. In addition, the economic burden has been calculated as greater than 3% of GDP [12]. In an effort to mitigate the impact of tobacco on its population, Bangladesh ratified the WHO Framework Convention on Tobacco Control (FCTC) in 2004 and passed the “Smoking and Using of Tobacco Products (Control) Act 2005”, subsequently updated and supplemented by the “Smoking and Using of Tobacco Products (Control) (Amendment 2013) Act 2005”. The Acts created smokefree indoor public places and transportation, restricted tobacco advertising, provided graphic health warnings on tobacco packaging and banned sales to and by minors. The Ministry of Health and Family Welfare was also mandated to create a National Tobacco Control Cell (NTCC) to oversee and guide implementation and enforcement.

The NTCC commenced Tobacco Control Task Force development in May 2007 ‘for effective enforcement of the Tobacco Control Act…The district and sub-district task force committees are the bodies entrusted with the task of tobacco control including enforcement of law under their jurisdiction’ [14] with the ultimate goal of reducing consumption by lowering the demand and supply for tobacco. The national level taskforce focuses primarily on policy activities, developing guidelines, information resources, measures to encourage tobacco crop substitution, and mechanisms for sustainable funding for tobacco control [15].

Local level taskforces are accountable to the NTCC and were designated to operate at district (zila) and sub-district (upazila) levels to enforce tobacco control laws within their jurisdiction. The creation of the local taskforces to an extent reflects the country’s governance structure whereby national laws are implemented at local level within 7 administrative divisions, 64 districts and 489 sub-districts. The challenging general context for law enforcement of a large population and a high level of illiteracy (over 50%, 2010 figures) [16] necessitates devolved implementation and underpinning by strategies to create public awareness. The concept is to develop a committee in each administrative area that will collectively plan tobacco control action, co-ordinate the relevant resources, undertake enforcement using mobile courts, and organise public information/awareness-raising activities. Mobile courts are a notable feature of the Bangladesh legal system; these courts are dispatched when authorities receive report of a violation and can try a case immediately at the location. Members of the public, organisations and officials can report any violation to the authorities for a mobile court then to pursue. This mechanism is used to enforce a range of laws, for example on food hygiene and trading standards. As regards tobacco law enforcement, these courts prosecute violations such as smoking or allowing smoking in public places and displaying tobacco advertising. The courts administer fines and remove illegal tobacco advertising [14]. The public trying of cases attracts public interest and consequently serves an important public education function, for example increasing awareness about the law and its rationale, such as the harm caused by tobacco [17]

Local taskforces were designed to have broad membership including representatives from the various enforcement authorities (including administrative, police and health departments) and civil society organisations engaged in tobacco control or with an interest in addressing tobacco on behalf of a segment of the population, who are nominated by the local government administration and invited to participate [17]. Besides serving the practical purpose of facilitating co-ordination and collaboration among stakeholders, this feature is also a function of wider governance reforms in Bangladesh introducing mandatory mechanisms for citizen participation [18].

Data on the total number of taskforces were not available at the time of this study, although the NTCC was developing a data collection system to commence in late 2014. Data are available on districts that have held taskforce meetings. In quarter two of 2014, this included 43 out of 64 districts (67%) [19]. These figures, however, are merely an approximation as it is not known if other areas have taskforces who did not meet in the recorded quarter and there is no indication whether the taskforces are fully operational or just being established.

**Figure 1:**
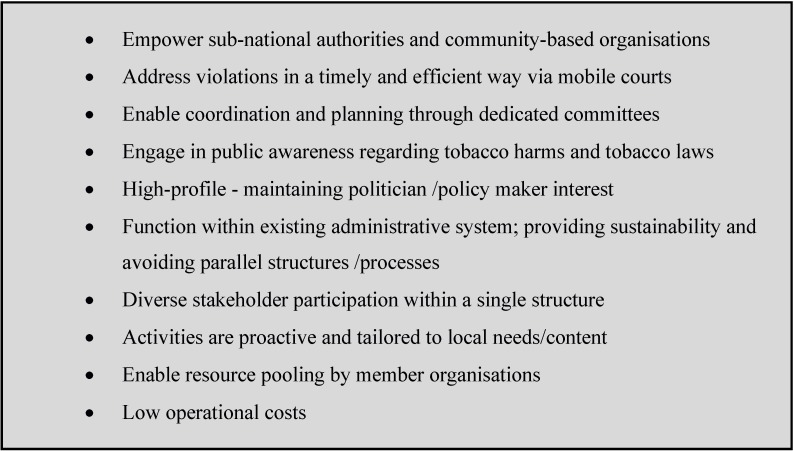
Key features of local Tobacco Control Taskforces.

A number of characteristics of the Bangladesh taskforces are seen in the tobacco control implementation approaches of other countries; however, the combination of features of the Bangladesh taskforce model, described in Figure 1 below, appears to be unique. For example, Indonesia has mobile courts that conduct random inspections, but these do not operate within the context of a national or sub-national body. In Pakistan, there are Provincial and District Implementation Committees that include a wide range of stakeholders, yet these committees do not use mobile courts as a key enforcement tool. And in Russia, the national tobacco law (2013) empowers sub-national administrations and community-based organisations but there are no dedicated multi-stakeholder committees for implementation or mobile courts for on-the-spot enforcement.

The Bangladesh local taskforce model appears to constitute a distinctive model of tobacco control implementation. The lack of research and data on this novel structure and the extent of their functioning and current contribution to tobacco control in Bangladesh prompted this study.

## 2. Methods

The aim was to gain insight into, and understanding of, district tobacco control taskforces—their current role, functioning, strengths, weaknesses and potential contribution to implementing tobacco control in Bangladesh, particularly from local perspectives. Interviewing key informants using an open-ended question schedule was selected as the most appropriate research method. This method allows access to individual perspectives and is open to the issues and opinions they may opt to take (emergent issues), yet also retains a strong focus on the particular topic areas central to answering the research questions [20,21]. This focused yet open-minded approach was considered appropriate and pragmatic, allowing an exploration of the issues on a topic on which little published research exists.

### 2.1. Participant Identification

A purposive strategy was applied to identify potential ‘key informants’ in each of the ten districts where taskforces were known to be operational. The aim was to engage a diverse range of individuals, with particular emphasis on those directly involved with the district taskforces (insider perspectives), while also comparing these findings with a smaller sample of non-involved local parties with an interest in tobacco control enforcement (outsider perspectives). Potential taskforce member interviewees were identified from public taskforce committee records. Potential non-taskforce member interviewees were identified via local contacts and information from local authority departments regarding individual officers and independent local associations with interests related to tobacco control implementation, for example retail and transportation associations. The selection strategy purposely sought to involve people from various local organisations and agencies with differing remits across the ten districts in order to represent a wide range of perspectives and experiences. Therefore, the interview data would be expected to encompass the range of major issues, themes, and interpretations one might find across the population of interest [22,23]—*i.e.*, among district taskforce members and a range of non-members.

Potential interviewees were contacted and provided with information about the study and ethical provisions such as the confidentiality of their interview and the anonymisation of data. Ethical approval for the study was obtained from the Scientific Committee of The International Union Against Tuberculosis and Lung Disease. The fact that the interviewers had previously met some interviewees in the course of their work and the interviewers’ disclosure of their own remit for tobacco control may have encouraged participation. Flexible scheduling and an option of telephone interviews if a face-to-face option was difficult to schedule may have also contributed. All invitations were accepted.

### 2.2. Participant Profile

Table 1 illustrates the profile of interviewees by district and organisation/remit. The total number of interviewees was 70, comprising 54 taskforce members (77%) and 16 non-members (23%).

Taskforce member interviewee selection varied depending on the composition of each local taskforce. In all ten districts, member interviewees included a senior local government administrative official, a senior police representative, the Women’s Officer and a senior health official; the majority also included a non-governmental organisation (NGO) representative. In a number of districts, additional member interviewees included retail/commerce organisation representatives and a legal officer member in one district. In all ten districts the non-member interviewees included the District Information Officer. Other non-member interviewees included shopkeepers and representatives from transport trade unions, commerce and retail associations and transport owners associations.

**Table 1:** Profile of interviewees by district and organisation/remit.

District	1	2	3	4	5	6	7	8	9	10		Total
*Interviewees (n)*	6	8	9	8	6	6	5	7	8	7		70
*Taskforce Members:*												
Senior Government Administrative Official	1	1	1	1	1	1	1	1	1	1		10
Senior Legal Official			1									1
Senior Health Official	1	1	1	1	1	1	1	1	1	1		10
Senior Police	1	1	1	1	1	1	1	1	1	1		10
Retail/Commerce Organisation		1	1	1				1				4
Women’s Affairs Officer	1	1	1	1	1	1	1	1	1	1		10
NGO representative	1	1	1	1	1	1		1	1	1		9
*Non-members:*												
Shopkeeper/owner									1	1		2
Transport Owners Association				1					1			2
Transport Union		1	1									2
District Information Officer	1	1	1	1	1	1	1	1	1	1		10

### 2.3. Data Collection

Interviews with the 70 participants were undertaken in Bengali over a period of four months in 2014. The majority of interviews (94%) were undertaken face-to-face and were held at participants’ work offices. A small number (6%) of interviews were undertaken by telephone if scheduling proved difficult for participants. A semi-structured interview guide with open-ended questions was used in all interviews to obtain interviewees’ perspectives relating to:

- Their relationship to/involvement with/awareness of the local taskforce
- Awareness of activities undertaken by local taskforce
- Perceived impact of current activities
- Perceived strengths and weaknesses of current taskforce operations
- Proposals for ways to strengthen/enhance taskforce impact
- Perception of taskforce potential contribution to wider tobacco control goals

Full notes were taken during and following interviews. Each interview was allocated a participant code to anonymise the data.

## 3. Analysis

Interview notes were translated into English by the lead interviewer to enable shared analysis by the research team. All interview notes were initially read and re-read by each team member to provide an overview of the data. The team used a pragmatic analytical approach combining induction and deduction [20], seeking themes that would answer the research questions under consideration [21] while also being alert for any ‘emergent themes’. Themes and sub-themes and patterns were identified by team members individually and then cross-checked, collectively discussed and agreed to provide analytical consensus.

## 4. Findings

### 4.1. A crucial Tool for Tobacco Control—Actual and Potential

Taskforces were universally perceived to be a crucial tool for tobacco control in Bangladesh. This view was consistent across all ten districts and across the range of members and non-members. There was, however, a marked and fundamental difference relating to the taskforces’ different stages of development and levels of functioning.

In five districts, taskforces were described as relatively well-established and fully functioning. They were meeting regularly, involving a range of stakeholders and undertaking key activities relating to enforcement of smokefree and advertising regulations and public awareness campaigns. Members and non-members alike described their taskforce as undertaking an important role and contributing valuably to local tobacco control outcomes.

‘Taskforces are playing [a] vital role to implement the TC law. It creates public awareness about the harmful effect of tobacco. Committee members meet regularly and jointly take the decision to enforce the law.’

(Member, District 8)

‘Undoubtedly taskforces can play a dynamic role to implement the tobacco control law.’

(Non-member, District 9)

In five districts taskforces were described as not yet well-established and/or not functioning properly. Interviewees indicated that these taskforces were not meeting regularly and were perceived to undertake few implementation/enforcement activities. Some members and non-members expressed frustration that this was the case, yet notably all interviewees in these districts still viewed the taskforce concept positively. Their positive perceptions were primarily about the potential role of taskforces and the issues they believed they would address rather than a reflection of their local taskforce in its present state.

‘Taskforce committee [is a] good strategy to implement the law, but this committee does not work properly.’

(Member, District 1)

‘If the taskforce is properly activated, certainly it will bring changes in tobacco control.’

(Non-member, District 5)

### 4.2. Awareness of Taskforces and Their Work

Taskforce members who worked in local government initially became aware of their local taskforce’s development via the official government notification circulated to all district authorities. Other members learned about their local taskforce via tobacco control meetings held by the authorities and/or NGO networks, or when invited to join the taskforce itself.

‘I came to know about the taskforce by attending meetings organised by the health department.’

(Member, District 1)

In districts where taskforces were more established, the majority of non-member interviewees were aware of their local taskforce. They received information via several channels. Officials often received the notification about taskforce establishment. Other stakeholders had observed or heard about taskforce activities conducted in their district and/or received information disseminated by its member organisations or the tobacco control network.

‘[A] few months back I came to learn about [the] taskforce while they conducted a rally as part of a tobacco control campaign.’

(Non-member, District 7)

The major difference in districts where taskforces were less well established was that few non-members were aware of the local tobacco control taskforce.

### 4.3. Taskforce Outputs and Impact—The Contribution to Local Tobacco Control

In the five districts where taskforces were described as established and functioning, members and non-members consistently highlighted positive impacts of their taskforce’s work. Some interviewees acknowledged that the taskforce was not solely responsible for these outcomes, as various agencies and organisations undertook activities individually as well as contributing to efforts as part of the taskforce. Nonetheless, interviewees felt strongly that the taskforce was an important part of the results they observed.

In these districts, members and non-members perceived that the taskforce activities had contributed to better enforcement of the law. All interviewees believed that smoking in public places and public transport had been reduced due to taskforce activities, particularly the mobile courts and public information. Reduced local tobacco advertising was also commonly reported as an important result of taskforce efforts, although to a slightly lesser extent than the perceived reduction in smoking in public places/transport. This was explained as being a more recent addition to taskforce enforcement after the law amendment in 2013 banned advertising.

‘Through mobile courts, law implementation is ensured and smoking in public places and public transport has been reduced.’

(Member, District 5)

‘Non-smokers are aware of their rights against smokers.’

(Non-member, District 7)

‘Tobacco advertisement has been totally banned [in] our district.’

(Member, District 8)

Increased public awareness about the harms of tobacco use and the national tobacco control law was commonly reported by interviewees in these districts. They linked this directly to the taskforce’s contribution to increasing public awareness and information activities.

‘People know about the harmful effect of using tobacco products.’

(Member, District 2)

‘Public awareness has increased through [the taskforce] installing billboards and signage regarding the tobacco control law.’

(Non-member, District 1)

Interviewees believed the taskforces have produced a valuable additional benefit, focusing attention on tobacco control as an issue of public importance and requiring the attention of the authorities.

‘Before, the district administration didn’t take [tobacco control] seriously. They were not interested to conduct mobile courts.’

(Member, District 8)

In the districts where the taskforces were not yet well established, a number of member interviewees believed that the limited activities that were starting to be undertaken—mainly the mobile courts—were contributing to reducing smoking in public places and increasing public awareness. Nonetheless, most members and non-members in these districts did not yet observe tangible benefits. The perceived potential benefits were highly similar to those cited in districts where taskforces were functioning better. Improved smokefree compliance, reduced smoking, removal of tobacco advertising, and improved public awareness of harms and the law were considered realistic expectations. Members and non-members emphasised the key proviso of the need to address the existing limitations in order for the taskforce to deliver the impacts they believed were possible.

### 4.4. Intensifying Activity and Improving Functioning

A strong and unifying theme across all ten districts was the need for taskforces to develop and improve, even those that were relatively longer established and functioning. In districts where taskforces were functioning relatively well, interviewees wanted them to augment their co-ordination functions and expand activities already underway.

‘Co-ordination among the committee members should be increased.’

(Member, District 4)

‘More mobile courts should be conducted and exemplary punishment should be given to the violators.’

(Non-member, District 9)

In districts where taskforces were said to be weak, interviewees emphasised the need to establish basic operations: meeting regularly, ensuring member attendance and planning more co-ordinated core activities such as mobile courts and awareness campaigns.

‘[Taskforces are] indeed a very effective step to reduce the tobacco burden but taskforce needs to implement the activities efficiently as directed in the notification.’

(Member, District 1)

A frequently cited recommendation was for the national authorities to monitor the taskforces. It was believed that this would address current deficiencies and develop stronger local delivery in districts that were not yet functioning well.

‘A reporting system can be established to ensure regular meeting.’

(Member, District 6)

‘Ensure taskforce accountability to NTCC.’

(Member, District 2)

In the less developed taskforce districts, interviewees also highlighted a need to increase and diversify taskforce membership to enable effective functioning.

‘[The] taskforce may involve more members including District Information Officer, public representatives…’

(Member, District 7)

Once these basics were addressed, the priority for all interviewees was to augment the taskforces’ local tobacco control efforts overall by planning and co-ordinating the efforts of various stakeholders. Specifically, respondents wanted intensive mobile court programmes and public awareness initiatives.

‘I would work to increase awareness about the harmful effect of tobacco and ensure huge publicity on it…installing billboards…schools programmes, meetings...’

(Member, District 1)

‘I would arrange more frequent mobile court operation.’

(Non-member, District 6)

### 4.5. Wider Actions Required to Support Local Progress on ‘MPOWER’ Areas

The priorities for local taskforce development were overwhelmingly those described above: improving functioning, enforcing the law on smokefree public places and advertising and building stronger public awareness (the ‘Protect’, ‘Enforce’ and ‘Warn’ areas of ‘MPOWER’). Nonetheless, a sizeable number of members and non-members whose work related to tobacco control highlighted wider actions that they considered essential to address the tobacco problem. The pressing need to address tobacco cultivation and livelihood substitution for tobacco farmers was most frequently raised. Interviewees suggested local as well as national action was required, with taskforces being well placed to address the issue locally. Need for structural actions at national level to control bidi production (locally made cigarettes), address pricing to reduce consumption and enable licensing of tobacco sellers were also proposed.

‘Supply side, like tobacco cultivation, availability of tobacco products in cheaper rate…has to be addressed’

(Member, District 6)

‘Stop providing loan[s] from all government and private banks and NGOs for cultivating tobacco...’

(Member, District 8)

## 5. Limitations

This qualitative study provides insight into the functioning and limitations of local taskforces as perceived by a sample of 70 members and non-members across ten districts. The findings additionally indicate areas of perceived impact and suggest thereby that well-functioning district taskforces can make an important contribution to tobacco control at district level. Evaluation studies including quantified measures, such as compliance surveys, air quality monitoring, local authority and court record data, are needed to confirm these findings and to consider the relative contribution of taskforces in local contexts where separate activities by different organisations are undertaken, and where external issues such as different levels of tobacco cultivation and tobacco industry activities may create more conducive or more difficult circumstances for law implementation. An additional limitation is that this study focused exclusively on district-level taskforces and thereby did not examine the perspectives of ‘national taskforce’ members and national-level non-member officials. These wider perspectives, benefitting from an overview of developments across the country, may be a helpful element to include in a future study.

## 6. Discussion

The findings suggest that when district tobacco control taskforces function properly, they can make an important contribution to tobacco control outcomes at the local level. Taskforce activities to promote and enforce the tobacco control law were seen by both members and non-members in half of the study districts (five) as making a valuable contribution to reducing smoking in public places and reducing tobacco advertisements. The difference in the way tobacco control impacts were discussed—as ‘actual’ impacts in districts where taskforces were working quite well, to impact being largely ‘potential’ in districts where the taskforce was not yet working well, lends credence to the indication that district taskforces are contributing to the effective implementation of key ‘MPOWER’ strategies, particularly ‘Protect’, ‘Warn’ and ‘Enforce’.

Half of the district taskforces in the sample, however, were described as not yet functioning well enough to be able to achieve notable results. These taskforces were considered a “work in progress” requiring further local commitment and additional management and support to achieve their potential.

The variation in stages of development and functioning of local taskforces has been recognised at national level. In autumn 2014, the NTCC was developing plans to address these issues with activated taskforces, and to introduce mandatory reporting and reiterate local authorities’ responsibility to ensure that taskforces function well. Additionally, and supporting this study’s finding of a strong perceived contribution of well-established district taskforces to local tobacco control outcomes, the NTCC also has plans to expand taskforce establishment to all districts and sub-districts across the country [19].

National meetings for taskforce representatives have been organised by NTCC since 2008. In the activated districts, tobacco control taskforces are seen to be delivering impacts and viewed as an important implementation tool; it is possible that representation of these views and examples at national meetings may partly explain the positive expectations in the districts where the taskforces were not yet fully functioning. Certainly, sharing good practice nationally would seem an excellent way to assist districts requiring development.

The ‘package’ of notable features of Bangladeshi local tobacco control taskforces appears to constitute a distinctive yet flexible model of tobacco control implementation. The breadth of stakeholder representation and combination of a co-ordination function with active law enforcement via mobile courts and public awareness development is apparently a unique tobacco control structure. Where taskforces were considered to be functioning well, the membership tended to comprise a more diverse range of stakeholders, including civil society representatives from, for example, NGOs and business/commerce organizations. This broader profile may have resulted from the better functioning taskforces having made greater efforts to develop this profile. Yet it is equally possible that the act of involving a more diverse profile of stakeholders from the outset may itself have contributed to the better functioning of these taskforces, for example by civil society members holding the local authority representatives to account. To capitalise on this successful element during the further expansion of local taskforces it is suggested that all taskforces should institute a formal code among their members to apply FCTC Article 5.3 [24]—to protect public policy from tobacco industry interference.

A particularly attractive feature of this model for LMICs is the low operational cost of taskforce functioning. This relates partly to the fact that taskforces operate within the existing administrative system, simply drawing together stakeholders to co-ordinate and enhance their collective output. This can thereby provide a sustainable mechanism for local tobacco control. On the issue of sustainability, the low operational costs are important, although interviewees did suggest that additional resources would be necessary in order to intensify activities, particularly public awareness-raising. This need could potentially be met if the NTCC is successful in establishing a mechanism for sustainable tobacco control funding, for example The National Board of Revenue introduced 1% health tax on cigarettes in the national budget of 2014—2015. A percentage of this fund could be allocated to the strengthening of the capacity of taskforces [25]. Granting district taskforces the power to use revenues from local tobacco law violation fines is another option.

## 7. Conclusions

This study suggests that the current and potential contribution of tobacco control taskforces to addressing tobacco at local level is substantial. It also highlights the need to build capacity in districts where taskforces are less established. The taskforce concept and lessons from the development process may provide other countries seeking a flexible local tobacco control implementation structure with a helpful model. Importantly, however, study participants believed that certain issues, especially tobacco cultivation and production, require national leadership and without this taskforces’ local efforts and impact would be stymied. Closer coordination between the national level taskforce and local taskforces could potentially lead to improved effectiveness and impact.
